# Decreased resting-state neural signal in the left angular gyrus as a potential neuroimaging biomarker of schizophrenia: An amplitude of low-frequency fluctuation and support vector machine analysis

**DOI:** 10.3389/fpsyt.2022.949512

**Published:** 2022-08-25

**Authors:** Yujun Gao, Xin Tong, Jianxiu Hu, Hanjun Huang, Tian Guo, Gang Wang, Yi Li, Gaohua Wang

**Affiliations:** ^1^Department of Psychiatry, Renmin Hospital of Wuhan University, Wuhan, China; ^2^School of Mental Health and Psychological Science, Anhui Medical University, Heifei, China; ^3^Wuhan Mental Health Center, Wuhan, China

**Keywords:** amplitude of low-frequency fluctuation, schizophrenia, resting-state fMRI, support vector machine, imaging biomarker

## Abstract

**Objective:**

Schizophrenia (SCH) is primarily diagnosed based on specific clinical symptoms, with the lack of any objective SCH-related biomarkers often resulting in patient misdiagnosis and the underdiagnosis of this condition. This study was developed to assess the utility of amplitude of low-frequency fluctuation (ALFF) values analyzed *via* support vector machine (SVM) methods as a means of diagnosing SCH.

**Methods:**

In total, 131 SCH patients and 128 age- and gender-matched healthy control (HC) individuals underwent resting-state functional magnetic resonance imaging (rs-fMRI), with the resultant data then being analyzed using ALFF values and SVM methods.

**Results:**

Relative to HC individuals, patients with SCH exhibited ALFF reductions in the left angular gyrus (AG), fusiform gyrus, anterior cingulate cortex (ACC), right cerebellum, bilateral middle temporal gyrus (MTG), and precuneus (PCu) regions. No SCH patient brain regions exhibited significant increases in ALFF relative to HC individuals. SVM results indicated that reductions in ALFF values in the bilateral PCu can be used to effectively differentiate between SCH patients and HCs with respective accuracy, sensitivity, and specificity values of 73.36, 91.60, and 54.69%.

**Conclusion:**

These data indicate that SCH patients may exhibit characteristic reductions in regional brain activity, with decreased ALFF values of the bilateral PCu potentially offering value as a candidate biomarker capable of distinguishing between SCH patients and HCs.

## Introduction

Schizophrenia (SCH) is a psychological disorder characterized by progressive changes in brain function that result in symptoms including reduced social function, decreased motivation and emotion, hallucinations, and delusions (1). Currently, SCH diagnoses are primarily made based on the symptoms and clinical signs that patients exhibit (2). As other psychiatric conditions including bipolar disorder and major depressive disorder (MDD) can exhibit symptoms similar to those of SCH, this can often result in patient misdiagnosis, underscoring the need for the establishment of objective biomarkers capable of aiding in SCH diagnostic efforts.

Resting-state functional magnetic resonance imaging (rs-fMRI) has emerged as a powerful tool that offers a potential means of identifying novel, sensitive biomarker signatures associated with specific brain disorders (3–6). A growing body of evidence suggests that abnormal changes in patients with SCH are primarily found in the striatum (7), temporal lobe (8), default-mode network (DMN) (9), and frontoparietal network (10), although the specific nature of these changes has varied across studies, with some reporting functional signal increases (11–13), decreases (14), or both (15). These discrepant findings may be attributable to the differences in sample size, disease course, and the analytical methods employed (16,17). Researchers have focused on these regional brain abnormalities when seeking to define biomarkers of SCH (17,18). Li et al., for example, utilized volumetric decreases in the left insula as a potential diagnostic endophenotype for SCH (19), while others have reported early decreases in global-brain functional connectivity in the bilateral anterior cingulate cortex (ACC) as a promising predictor of SCH patient therapeutic outcomes (20). In a recent article, Li et al. established a novel hypothesis-driven neuroimaging biomarker of SCH through a comparison of these patients and healthy control (HC) individuals, achieving > 80% accuracy (17). However, their developed biomarker necessitated the integration of several functional indicators, making it impractical for routine or urgent clinical use in a diagnostic or therapeutic setting. Ideally, a biomarker of SCH should be readily obtained, non-invasive, and associated with a high degree of diagnostic accuracy, although no such biomarkers have yet been identified despite decades of intensive research (21).

The amplitude of low-frequency fluctuations (ALFF) in the BOLD signal measured during rs-fMRI analyses can provide insight into spontaneous brain functional activity, and it is thus commonly used when evaluating patients with diseases including cervical spondylotic myelopathy (22), SCH (17), and depression (23). ALFF values have been shown to offer great promise as diagnostic biomarkers owing to their high degree of high temporal stability. Using an ALFF approach, SCH showed some abnormalities in spontaneous brain activity in parietal and occipital lobes (24). Furthermore, Kirino et al. (15) determined that SCH patients exhibited changes in spontaneous brain activity in two separate frequency bands. In addition, a large number of previous studies have found that the age of publication, the course of the disease, and the drug have some effects on brain function. Therefore, we selected patients with SCH with an earlier age of onset and a shorter course of disease as the study subjects to reduce confounding factors in this regard.

The application of support vector machine (SVM)-based artificial intelligence methods has been increasingly used to aid in predicting therapeutic outcomes or accurately diagnosing specific conditions (25,26). Multivariate pattern recognition-based SVM methods allow for the detection of patterns within a given dataset, and are well-suited to analyzing high-dimensional data in which there are more features than there are observations, as is common in experimental settings (27). SVM approaches enable optimal hyperplane separation in high-dimensional space, with samples closest to this hyperplane being defined as support vectors. When performing fMRI studies, SVM weights can be overlapped with the original brain space to generate a discriminative map by visually tracing the most important weights to the regions of the brain with the most discriminative value. SVM strategies have been shown to offer great clinical utility in the context of high-dimensional neuroimaging data-based decision-making (28,29). Here, rs-fMRI data from SCH patients were examined and ALFF values from abnormal regions of the brain were extracted and evaluated for their potential utility as neuroimaging biomarkers of SCH through the use of SVM methods. Together, the results of this study have the potential to aid in the more reliable and efficient diagnosis of SCH.

## Materials and methods

### Subjects

For this analysis, 131 patients diagnosed with SCH and 128 age- and gender-matched HC individuals were consecutively recruited from the inpatients or outpatients of the Wuhan Mental Health Center and Renmin Hospital of Wuhan University. Patients were diagnosed with SCH in accordance with the criteria established in the Diagnostic and Statistical Manual of Mental Disorders—Fourth Edition (DSM-IV). Prior to screening, all study participants were assessed with the Chinese MINI version of the Concise International Neuropsychiatric Interview, and two psychiatrists with different professional titles independently diagnosed all patients. While 5 participants (2 HCs, 3 SCH patients) exhibited excessive head movements during initial imaging, they were rescreened on the same day such that no data needed to be excluded from this study. All enrolled SCH patients experienced auditory hallucinations, reference delusions, persecutory delusions, and were 18–64 years of age. Participants were excluded from this study if they exhibited a history of substance abuse, electroconvulsive therapy, left-handedness, neurological disease, or severe illness. Prior to scanning, SCH symptoms were assessed based upon the Chinese versions of the Positive and Negative Symptom Scale (PANSS) and the Repeatable Battery for the Assessment of Neuropsychological Status (RBANS) before scanning (30,31). After scanning, patients were successfully followed for 3 months and the diagnosis of SCH was confirmed. HC participants were recruited from universities and the community, were free of any history of severe medical or neuropsychiatric illnesses, and did not exhibit any family history of neuropsychiatric disease among first-degree relatives.

### Magnetic resonance imaging scanning procedures

Philips Ingenia 3.0 T scanners in the Mental Health Center of Wuhan Affiliated with Huazhong University of Science and Technology were used to conduct rs-fMRI scanning for all study participants. Participants were directed to close their eyes, remain awake, and avoid thinking about anything in particular to the greatest extent possible. Functional images were captured using a gradient-echo—echo-planer imaging sequence to acquire data with the following settings: TR/TE = 2,000 ms/30 ms, thickness (mm) = 60, 35 slices, 64*64 element matrix, a flip angle of 78, 22.4 cm field of view, 3.5 mm slice thickness, 0.6 mm gap and 1 mm pitch, total scan duration“09:17.7.” 3D_T1 scanning parameters: repetition time (TR) = 8.4 ms, echo time (TE) = 3.2 ms, slice thickness = 1 mm, slice spacing = 0 mm, Number of slice = 33, and field of view (FOV) = 256 × 256 cm, total scan duration“04:17.7.”

### Data processing

The pre-processing of rs-fMRI data was conducted in Matrix Laboratory (MATLAB) using Data Processing Assistant for Resting-State fMRI (DPARSF). The impact of initial signal instability on the resultant analyses was reduced by discarding the first 10 time points. Data were corrected for head movement and slice time. Any participants that exhibited > 2 mm maximum displacement in the x-, y-, or z-axis or > 2° maximum rotation underwent rescanning on the same day until meeting these criteria. Corrected imaging data were subjected to spatial normalization to the T1 imaging and standard Montreal Neurological Institute space. The resultant images were then resampled at 3 × 3 × 3 mm^3^, band-pass filtered (0.01–0.08 Hz), and linearly detrended. Spurious covariates were eliminated, including the signal from a region centered in the white matter and the signal from a ventricular seed-based region of interest. The resultant data were then smoothed using a Gaussian kernel of 6 mm full-width at half-maximum.

### Amplitude of low-frequency fluctuations analyses

ALFF analyses were proposed by Jia et al. (32), and were conducted in MATLAB with the REST software (33). ALFF values were based on measurements of the rs-fMRI signal for each voxel, and were sensitive to the scale of the raw signal. Time series data for each voxel were subjected to fast Fourier transformation into the frequency domain, with the power spectrum then being calculated and subjected to square root transformation for each voxel. Average square root values were measured as the ALFF across the 0.01–0.08 Hz range for each voxel, with the ALFF then being calculated.

### Statistical analyses

Differences in age, years of education, PANSS, and RBANS results were compared between SCH patients and HCs using two-sample *t*-tests, while chi-square tests were used to assess differences in gender distributions between these groups using SPSS 23.0. Age, gender, years of education, and frame-wise displacement were used as covariates. Correlations between abnormal ALFF values and clinical variables were assessed through Spearman’s correlation analyses. *P* < 0.05 was the threshold of significance. Differences between groups were identified through a voxel-by-voxel analysis of covariance using individual whole-brain ALFF maps for these two groups. Results were thresholded at *P* < 0.01 and GRF-corrected *via* cluster-extent-based thresholding with a primary threshold of *P* < 0.01 in REST.

### Classification and receiver operating characteristic analyses

SVM methods were implemented in MATLAB with a library for support vector machine (LIBSVM) software package. At first, two-sample *t*-tests were conducted to identify significant regions between the patient and control groups, and then SVM was used based on the ALFF values of the identified regions. A grid of parameters was evaluated using LIBSVM, with the accuracies of all parameter settings being acquired after which the highest cross-validation accuracy for these parameters was established (for more detailed procedures, see Supplementary Material). ROC curves were used to analyze the ALFF values in abnormal brain regions.

## Results

### Participants

In total, this study enrolled 131 patients diagnosed with SCH and 128 age- and gender-matched HCs. Participant clinical and demographic data are summarized in Table 1, revealing no differences among these groups with respect to age, gender, or years of education.

**TABLE 1:** Characteristics of the participants.

Demographic data	Patients (*n* = 131)	HCs (*n* = 128)	*T* (orx^2^)	*P*-value
Gender (male/female)	131 (76/55)	128 (75/53)	0.35	0.85^a^
Age (years)	26.34 ± 10.39	30.33 ± 7.90	3.04	0.08^b^
Education (years)	9.50 ± 2.64	9.30 ± 2.61	0.61	0.34^a^
Onset (years)	19.42 ± 12.21			
Illness course (years)	6.92 ± 4.86			
REBANS	177.61 ± 38.60			
PANSS	89.63 ± 17.00			

^a^The *p*-value for gender distribution was obtained by chi-square test.

^b^The *p*-value were obtained by two sample *t*-tests.

HCs, healthy controls; REBANS, Repeatable Battery for the Assessment of Neuropsychological Status; PANSS, Positive and Negative Symptom Scale.

### Schizophrenia-related differences in amplitude of low-frequency fluctuations values

**FIGURE 1:**
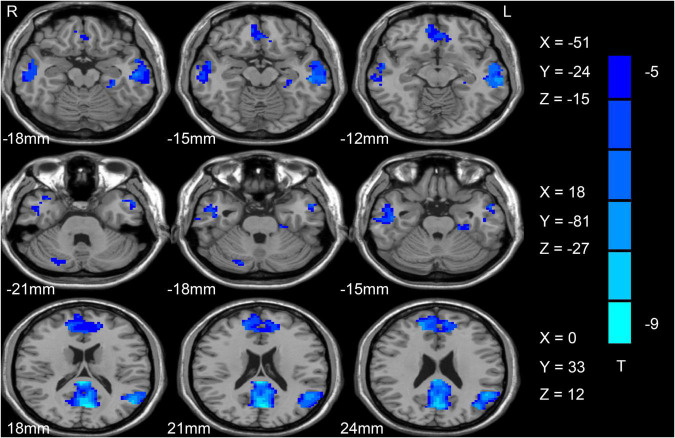
Differences in ALFF values between SCH patients and HC individuals. Reduced ALFF values are shown in blue, with the color bar representing *t*-values in the group analysis. ALFF, amplitude of low-frequency fluctuation; SCH, schizophrenia; HCs, healthy controls.

Initially, differences in ALFF values were compared between SCH patients and HC individuals using two-sample *t*-tests, revealing significant decreases in these values in the left angular gyrus (AG), ACC, fusiform, right cerebellum, bilateral precuneus (PCu), and middle temporal gyrus (MTG) in SCH patients relative to HCs (Figure 1 and Table 2).

**TABLE 2:** Brain regions with abnormal ALFF in schizophrenia patients.

Cluster location	Peak X	(MNI) Y	Z	Cluster size (voxels)	Peak accuracy (%)	*T*-value
**Patients < HCs**						
Right cerebellum	18	−81	−27	58	69.11	−7.38
Left MTG	−51	−24	−15	399	70.27	−7.91
Right MTG	60	−39	−3	246	70.27	−8.25
Left fusiform	−30	−33	−21	41	69.88	−7.41
Left ACC	0	33	12	148	70.66	−8.2
Bilateral PCu	−6	−72	18	273	73.36	−8.75
Left AG	−51	−54	24	460	73.36	−8.15

MNI, montreal neurological institute; MTG, middle temporal gyrus; ACC, anterior cingulate cortex; PCu, Precuneus; AG, angular gyrus.

### Support vector machine results

**FIGURE 2:**
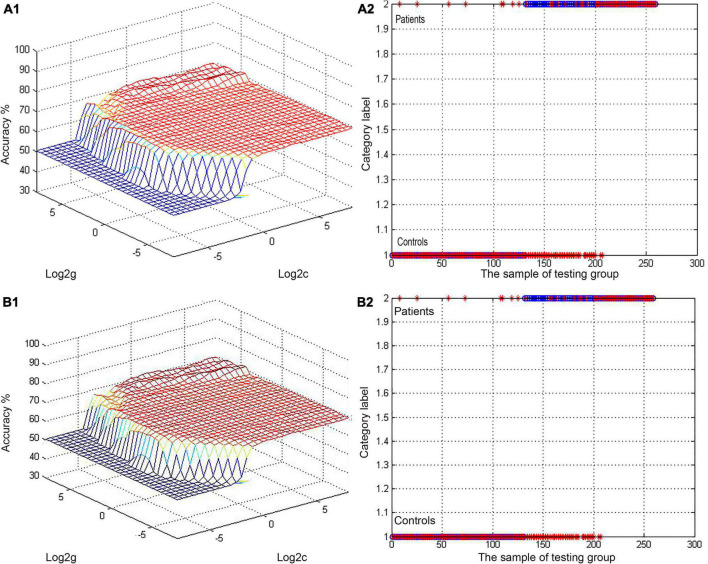
**(A,B)** Visualization of SVM classification based upon reduced ALFF values in the bilateral PCu and left AG as a means of differentiating between SCH patients and HCs. A1/2: 3D visualization of SVM with the most optimal parameters; B1/2: classification map of ALFF values for the bilateral PCu/left AG. SVM, support vector machine; ALFF, amplitude of low-frequency fluctuation; AG, angular gyrus; PCu, precuneus; SCH, schizophrenia; HCs, healthy controls.

**FIGURE 3:**
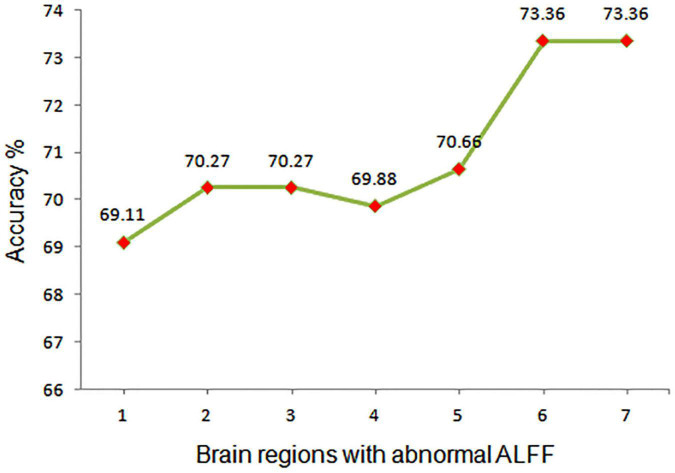
Assessment of the accuracy of utilizing abnormal ALFF values in different regions of the brain to differentiate between SCH patients and HCs. ALFF, amplitude of low-frequency fluctuation; 1, right cerebellum; 2, middle temporal gyrus; 3, right middle temporal gyrus; 4, left fusiform; 5, left anterior cingulate cortex; 6, bilateral precuneus; 7, left angular gyrus.

Next, abnormal ALFF values in different regions of the brain were used to classify individuals in these two groups. The bilateral PCu and left AG in SCH patients were individually analyzed using the SVM method, revealing that reduced ALFF values in the bilateral PCu could readily differentiate between SCH patients and HCs with good accuracy (73.36%), specificity (54.69%), and sensitivity (91.60%) (Figures 2A, 3). Similarly, decreased ALFF values in the left AG were capable of discriminating between these two groups of patients with satisfactory accuracy (73.36%), specificity (52.34%), and sensitivity (93.89%) (Figures 2B, 3).

### Receiver operating characteristic results

**FIGURE 4:**
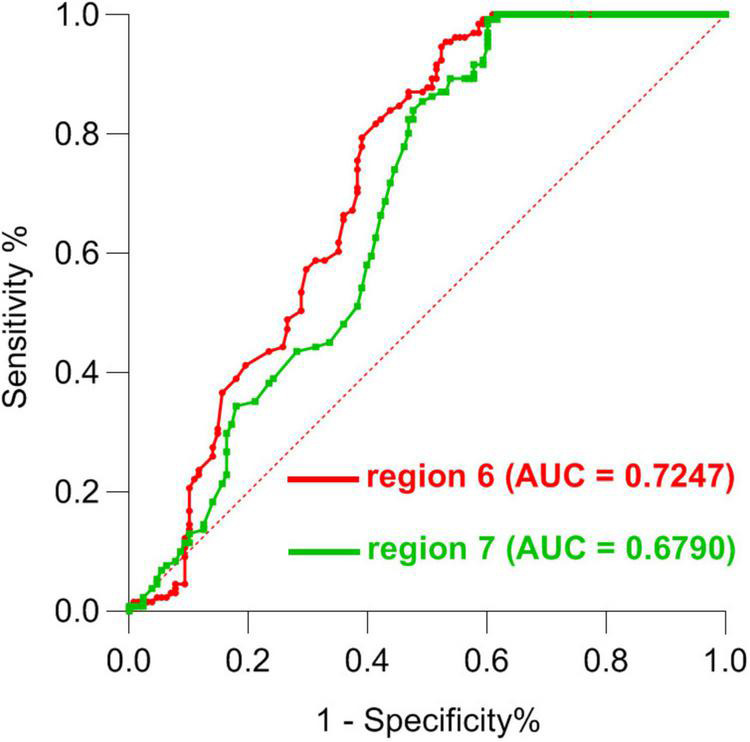
ROC curve-based differentiation between SCH patients and HC individuals based upon ALFF values in region 6 and region 7 of the brain. ROC, Receiver operating characteristic; SCH, schizophrenia; HCs, healthy controls; ALFF, amplitude of low-frequency fluctuation; 6, bilateral precuneus; 7, left angular gyrus.

**FIGURE 5:**
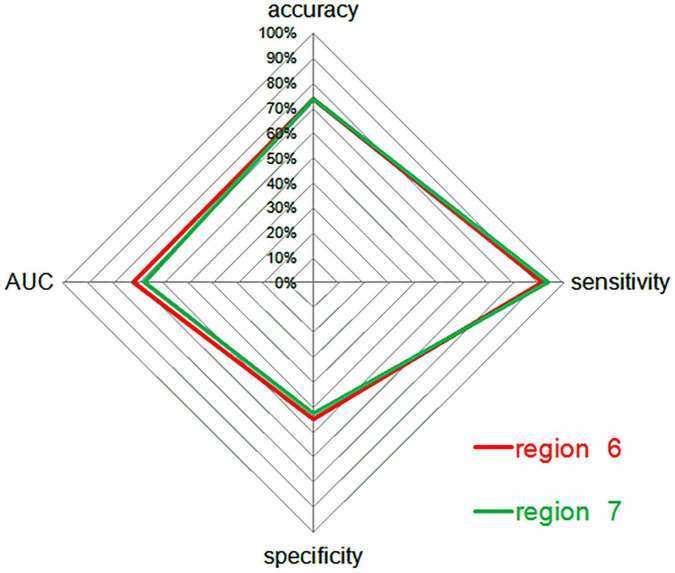
A radar plot demonstrating the accuracy, sensitivity, and specificity of classifications for SCH patients and HCs between region 6 and region 7, with corresponding AUC values. AUC, area under the curve; SCH, schizophrenia; HCs, healthy controls; 6, bilateral precuneus; 7, left angular gyrus.

Next, ROC curve analyses were employed as a means of comparing the accuracy for analyses of region 6 and region 7, revealing that ALFF values for these two regions could be effectively applied to differentiate between SCH patients and HCs while achieving good sensitivity and specificity. Through this analysis, abnormal ALFF values in the bilateral PCu were found to be superior as a candidate biomarker for distinguishing between SCH patients and HCs (Figures 4, 5 and Table 3).

**TABLE 3:** ROC analysis for differentiating SCH patients from HCs by using ALFF values in region 6 and 7.

Brain regions	Area under the curve	Cut—off point	Sensitivity	Specificity
Region 6	0.7247	1.160	91.60%	54.69%
Region 7	0.6790	1.295	93.89%	52.34%

ROC, receiver operating characteristic; SCH, schizophrenia; HCs, healthy controls; ALFF, amplitude of low-frequency fluctuation; 6, bilateral precuneus; 7, left angular gyrus; AUC, area under the curve.

### Correlations between amplitude of low-frequency fluctuations values and clinical variables

Abnormal ALFF values in the identified brain regions were not found to correlate with any clinical variables in this patient cohort.

## Discussion

Here, reduced ALFF values were observed in the left AG, ACC, fusiform, right cerebellum, bilateral PCu, and MTG of SCH patients relative to HCs. When ALFF values for these abnormal brain regions were utilized as candidate biomarkers to differentiate between SCH patients and HCs *via* an SVM approach, decreased ALFF values in the bilateral PCu were able to discriminate between these two groups (accuracy: 73.36%, specificity: 54.69%, sensitivity: 91.60%), as were decreased ALFF values in the left AG (accuracy: 73.36%, specificity: 52.34%, sensitivity: 93.89%). Subsequent ROC analyses indicated that abnormal ALFF values in the bilateral PCu may offer value as an SCH-related neuroimaging biomarker, with an AUC of 72.47%.

The PCu is a critical component of the DMN, which corresponds to a series of functionally consistent networked brain regions (the medial prefrontal cortex, posterior cingulate cortex/PCu, medial parietal cortex, lateral parietal cortex, inferior parietal cortex, and cerebellum) that exhibit high activity levels at rest (34), with activity levels being reduced when the brain is engaged in non-specific attention task execution. A growing body of evidence supports a close relationship between the DMN and mental activity (35,36), with abnormal network homogeneity having been reported in the DMN of drug-naïve, first-episode adolescent SCH individuals (37). Additionally, different SCH subtypes have been found to exhibit significant differences in resting-state DMN activity (38). Researchers have also found that there are similar situations in different types of other mental diseases. For example, Chen et al. Found that bipolar depression and MDD have common abnormal brain activity (increased dynamic functional connectivity variability between the left dorsal rostral putamen and the left supplementary motor area, and between the right VRP and the right inferior parietal lobe), Had specific increased dynamic functional connectivity variability between the right dorsal caudal putamen and the left central gyrus compared with MDD and HCs (39). A multimodal meta-analysis of resting-state studies showed that bipolar disorder was characterized by hypo-connectivity within the default network, the affective network, and ventral attention network etc., and decreased gray matter volume in the insula, inferior frontal gyrus, and ACC (40). Recently, increased functional connectivity was observed between the right PCu and cerebellum, temporal lobe, and left superior parietal lobule in non-depressed SCH individuals (41), while another study reported reduced ReHo in the right superior temporal gyrus, left middle frontal gyrus, PCu, and left central anterior gyrus in childhood- and adolescent-onset SCH, with auditory hallucination incidence being associated with abnormal activity in these regions (42). These inconsistent results may be attributable to differences in sample size, disease course, or analytical approaches employed among studies. Furthermore, structural MRI findings have suggested that a gray matter volume reduction was evident in the bilateral PCu in SCH patients (43). In one recent meta-analysis, reduced ALFF values within the PCu were found to be reliably associated with memory and theory of mind in both first-episode and chronic SCH patients (44). Moreover, damage to the PCu can impact frontal lobe activity (45). These prior studies suggest that abnormal ALFF values in the PCu, medial superior frontal gyrus, MTG, and illness severity are likely to be correlated with one another. Even so, no such correlative relationships were observed in the present study. Abnormal ALFF values in the DMN may be attributable to the sample size in this study or the specific characteristics of this patient population. Moreover, this analysis revealed that reduced ALFF values in the bilateral PCu may serve as a promising biomarker for differentiating between SCH patients and HCs in an SVM analysis, yielding good accuracy (73.36%), specificity (54.69%), and sensitivity (91.60%).

The AG is located in the posterior of the inferior parietal lobe, and serves as a central hub for different subsystems (46). The AG plays a role in semantic processing, reading, and word comprehension, and is linked to higher memory scores (47). Over the course of SCH progression, patients exhibit increases in brain damage, with chronic SCH patients exhibiting the disruption of normal bilateral AG asymmetry and a reversal of the normal left > right asymmetry relative to HC individuals (48). In this analysis, decreased ALFF values were only observed in the left AG of analyzed SCH patients, potentially owing to the shorter disease course in these individuals. The AG is considered a component of the semantic-lexical network that includes the planum temporal lobe and plays a role in complex thought processes and the perception of auditory hallucinations (49). Previous reports have documented abnormal cortical asymmetry in the superior temporal gyrus in individuals with SCH, particularly in the AG (50). In the present study, SCH patients were generally considered to exhibit memory loss and slower cognition as these traits are associated with reductions in ALFF values within the AG. These abnormal AG findings offer important insight into the neurological basis for the characteristics of thought and language processing in individuals diagnosed with SCH. When ALFF values in the left AG were used as a biomarker to differentiate between SCH patients and HCs *via* an SVM approach, the associated specificity (71.88%), sensitivity (93.89%), and accuracy (52.34%) values suggested that reduced ALFF values in the left AG may represent an effective biomarker of SCH. No correlations, however, were observed between these reduced ALFF values and SCH patient disease severity or course. This may be attributable to the fact that brain function is impacted by a range of factors including compensatory mechanisms, or may suggest that decreased ALFF in left AG is a unique intrinsic characteristic of SCH that does not impact clinical symptoms of this disease.

The MTG plays a critical role in regulating sensory perception and is generally regarded as a central region of the brain necessary for memory and language functions (51,52). Neuroimaging data from SCH patients have consistently revealed a role for this region of the brain in the context of hallucinations and emotional processing. In addition, the MTG serves as a significant node in the network consisting of the frontal lobe, temporal lobe, parietal-occipital lobe, and subcortical structures (53,54). Abnormal MTG activation may thus impair language processing and semantic memory functions. In this analysis, decreased ALFF in the bilateral MTG was observed in patients with SCH, and the resultant abnormalities may impair language and memory functions in these individuals, resulting in symptoms characteristic of this disease.

There are several limitations to this study. For one, some of the included SCH patients had been medicated prior to study initiation, and it is thus not possible to exclude the impact of such treatment on the observed structural and functional alterations in the brains of SCH patients (55). Subsequent studies of SCH patients that have not undergone drug treatment will be necessary to clarify this possibility. Second, this was a single-center study with a relatively small sample size, highlighting a need for additional large-scale multi-center validation of these results. Finally, deep learning is currently the most scientific in the exploration of biomarkers of brain function disorders in psychiatry. However. Our research only focuses on traditional SVM. In the next step, we plan to expand the sample size and collect schizophrenia data from multiple centers to further explore the imaging biomarkers of schizophrenia combined with artificial intelligence technology.

## Conclusion

Overall, the results of this analysis suggest that SCH patients exhibit abnormal ALFF values in the left AG, ACC, fusiform, right cerebellum, bilateral PCu, and MTG. In particular, the reduced ALFF values in the left AG may offer value as a candidate biomarker to guide the objective diagnosis of SCH.

## Data availability statement

The original contributions presented in this study are included in the article/Supplementary material, further inquiries can be directed to the corresponding author/s.

## Ethics statement

The study was approved by the ethics committee of the Wuhan Mental Health Center in accordance with the Declaration of Helsinki, and all subjects signed the written informed consent. The patients/participants provided their written informed consent to participate in this study.

## Author contributions

YG and XT conceived the structure of the manuscript and wrote the manuscript. GW, JH, HH, and TG collected and analyzed the data. YL and GW conceived and critically reviewed the manuscript. All authors have read and approved the final manuscript.
