# Supplementary material for: Decreased resting-state neural signal in the left angular gyrus as a potential neuroimaging biomarker of schizophrenia: An amplitude of low-frequency fluctuation and support vector machine analysis
Source: Front Psychiatry. 2022 Aug 25;13:949512. doi: 10.3389/fpsyt.2022.949512 (PMC9452648; doi:10.3389/fpsyt.2022.949512)
Supplement: Supplementary file 1 [file Presentation_1.pdf]

Decreased neural signal in left angular gyrus as a potential neuroimaging biomarker in schizophrenia patients at rest : An Amplitude of Low Frequency Fluctuation and Support Vector Machine Analysis

Yujun Gao[1#] PhD, Xin Tong MD[23#], Jianxiu Hu[3] MD, Hanjun Huang[3] MD, Tian Guo[3] MD, Gang Wang[3*] PhD, Yi Li[3*] PhD, Gaohua Wang[1*]

1 Department of Psychiatry, Renmin Hospital of Wuhan University, Wuhan, Hubei Province, China

2 School of Mental Health and Psychological Science, Anhui Medical University, Heifei, Anhui, China

3 Wuhan Mental Health center

# Yujun Gao and Xin Tong contributed equally to this work as a co-first author

* Corresponding author: Gang Wang, 532614206@qq.com, Yi Li, psyyee@gmail.com and Gaohua Wang, wanggaohua64@126.com

Support vector machine analyses

Support Vector Machine (SVM) is a popular machine learning method for classification. The main two steps included: first, training a dataset to acquire a model. Second, applying the acquired model to predict information of a testing dataset [1]. In the study, the method of "leave - one - out - test" was applied to validate the results [2]. Detailed procedures and package usage can be found on the website (https://www.csie.ntu.edu.tw/~cjlin/libsvm/): A classification task usually involves separating data into training and testing sets. Each instance in the training set contains one "target value" (the class labels) and several "attributes" (the features or observed variables). The goal of SVM is to produce a model (based on the training data) which predicts the target values of the test data given only the test data attributes. Here training vectors are mapped into a higher (maybe infinite) dimensional space by the function φ. SVM finds a linear separating hyperplane with the maximal margin in this higher dimensional space. Furthermore, the kernel function was used to analysze testing data. Linearly scaling features before applying SVM is very important. The main advantage of scaling is to avoid attributes in greater numeric ranges dominating those in smaller numeric ranges. Another advantage is to avoid numerical difficulties during the calculation. Because kernel values usually depend on the inner products of feature vectors. We use the same method to scale both training and testing data. A common strategy is to separate the data set into two parts, of which one is considered unknown. The prediction accuracy obtained from the "unknown" set more precisely reflects the performance of classifying an independent data set. An improved version of this procedure is known as cross-validation. In v-fold cross-validation, we first divide the training set into v subsets of equal size. Sequentially one subset is tested using the classifier trained on the remaining v−1 subsets. Thus, each instance of the whole training set is predicted once so the cross-validation accuracy is the percentage

of data which are correctly classified data. The cross-validation procedure can prevent the over fitting problem. A feasible approach for vast data sets is to choose a subset of the data set randomly, conduct a grid-search on them, and then do a better-region-only grid-search on the complete data set [3].

references

1. Chih-Chung, C. and L. Chih-Jen, *Libsvm: a library for support vector machines.* 2011.
2. Yi, L., et al., *Abnormal neural activity as a potential biomarker for drug-naive first-episode adolescent-onset schizophrenia with coherence regional homogeneity and support vector machine analyses.* Schizophrenia Research, 2017.
3. Chang, C.C. and C.J. Lin, *LIBSVM: A library for support vector machines.* ACM Transactions on Intelligent Systems and Technology, 2007. **2**(3, article 27).
